# Comprehensive first principles study on CO and NO gas adsorption effects on the structural, electronic, and optical properties of armchair silicon-tin nanoribbons

**DOI:** 10.1098/rsos.250927

**Published:** 2025-11-12

**Authors:** Tran Minh Tien

**Affiliations:** ^1^ Thu Dau Mot University, Vietnam

**Keywords:** ASiSnNR, gas adsorption, electronic band structure, optical properties, gas-sensing applications

## Abstract

This study presents a detailed first-principles investigation of the effects of CO and NO gas adsorption on the structural, electronic and optical properties of armchair silicon-tin nanoribbons (ASiSnNRs). Cohesive and adsorption energy calculations indicated that the ASiSnNR structure was thermodynamically stable, with physisorption for CO (−0.01 eV) and chemisorption for NO (−0.68 eV). Electronic band structure analysis revealed that pristine ASiSnNRs exhibited semiconducting behaviour with a narrow band gap (approx. 0.43 eV), which slightly widened upon CO adsorption and transitioned to a metallic state upon NO adsorption because of the strong orbital hybridization and charge transfer effects. Charge density and wave function analyses confirmed this mechanism, with particular emphasis on the role of the π* orbital of the CO molecule. The dielectric function, optical absorption, reflection spectra and joint density of states show significant enhancements in anisotropic optical properties after CO adsorption, especially in the ultraviolet region. These findings suggest the strong potential of ASiSnNRs for selective and highly sensitive gas-sensing applications, particularly for the detection of NO.

## 1. Introduction

Nanoribbon materials have attracted considerable research interest because of their promising applications in diverse fields such as nanoelectronics, optoelectronics and gas sensing. The earliest nanoribbons to receive intensive attention were graphene nanoribbons (GNR). The mechanical and thermoelectric properties of GNRs have been studied extensively since 2009 [1–3]. Faccio *et al.* investigated the mechanical properties, including Young’s modulus, Poisson’s ratio and shear modulus of zigzag GNR, along with the dependence of the electronic structure and mechanical response on strain [1]. Their results showed that GNRs possess a higher Young’s modulus than pristine graphene, suggesting enhanced stiffness owing to the reconstruction of the edge C–C bonds. Furthermore, the electronic structure exhibited minimal sensitivity to strain within the linear regime, indicating its great potential for nanoelectronic device applications.

The thermal conductivity of GNRs has also been explored [2], revealing exceptionally high values for long ribbons, which is consistent with the experimental findings for graphene. Notably, the edge configuration strongly affects the thermal transport; zigzag-edged GNRs (ZGNRs) exhibit higher conductivity than armchair-edged GNRs (AGNRs). The electronic and transport properties of GNRs were further elaborated by Wakabayashi *et al.* [3], who demonstrated that the edge type (zigzag versus armchair) significantly influences electronic behaviour. Zigzag edges generate localized edge states near the Fermi level, whereas armchair edges do not. These edge states support the emergence of perfectly conducting channels, which are crucial for efficient charge transport.

More general characteristics of GNRs have also been reported [4]. A notable finding is the direct correlation between the nanoribbon width and the band gap enlargement. Several fabrication methods have been developed, including graphene etching, chemical synthesis and carbon nanotube unzipping. Critical properties such as band gap tuning and charge carrier modulation have also been discussed [5].

In addition to GNRs, silicene nanoribbons (SiNRs) have attracted increasing attention [6–8]. Studies have shown that armchair SiNRs exhibit band gap oscillations that follow a periodic trend of 3*n* > 3*n* + 1 > 3*n* + 2 (where *n* is a positive integer). Different magnetic configurations (nonmagnetic, ferromagnetic and antiferromagnetic) significantly influence the band gap, resulting in either semiconducting or semimetallic behaviour. These structures display strong optical absorption at certain wavelengths, with excitonic effects playing a vital role in the optical transitions. Additionally, thermal properties such as electronic thermal conductivity and heat capacity increase with temperature owing to the thermal excitation of charge carriers into the conduction band.

Other types of nanoribbons, such as germanene, stanene and phosphorene, have also been actively investigated [9–11]. Germanene nanoribbons were found to possess direct band gaps at the Γ point, ranging from 0.0889 to 0.7528 eV, depending on the ribbon width, with a hybridization mix of sp² and sp³ bonds, where σ bonds are stronger than π bonds. For phosphorene nanoribbons, edge hydrogenation leads to semiconducting behaviour with larger band gaps than those of pristine phosphorene and a width-dependent quantum-confinement effect. In the case of stanene nanoribbons (SnNRs), a wide-band gap semiconducting nature is observed, which narrows with increasing ribbon width.

In addition to investigating the structural, thermal, electronic and optical properties of nanoribbon materials, gas adsorption on nanoribbon surfaces has also received increasing attention. Gas adsorption on graphene surfaces has been studied extensively [12–15]. Results show that AGNRs are highly sensitive to NH₃ adsorption, exhibiting n-type semiconducting behaviour. By contrast, gases such as CO, NO, NO₂, O₂, N₂ and CO₂ exerted a minimal impact on the conductivity of the GNRs. Among these, NH₃ and CO₂ displayed weak chemisorption, whereas CO, NO, NO₂ and O₂ tended to form stronger chemical bonds with the surface. CO, NO and CO₂ act as electron acceptors, whereas NH₃ serves as an electron donor. Notably, CO₂ adsorption transforms the AGNRs into p-type semiconductors. The adsorption of CO and CO₂ also leads to variations in the electrical conductivity of the GNRs, with different adsorption strengths contributing to different levels of conductivity modulation.

The gas adsorption behaviour of doped GNR has also been reported [16–19]. For instance, osmium doping significantly increased the CO adsorption energy on GNRs to −9.53 eV—approximately 43 times greater than that of pristine graphene. Additionally, B- and N-doped or defected GNRs show increased adsorption energies ranging from −0.19 to −0.87 eV. In the context of detecting NO-family toxic gases at trace concentrations using ZGNRs, results demonstrate high sensor reactivity (*S* = 4.63), approximately 21% selectivity for NO, and extremely short recovery times (103.00 and 0.01 µs), indicating excellent reusability and stability. Different gas molecules exhibit varying impacts on the electronic structure of AGNRs; among them, NH₃ is the only gas capable of modulating AGNR conductivity. CO₂ had the shortest recovery time, followed by CO, NO, and NH₃.

Gas adsorption has also been explored for a range of other nanoribbon materials including silicene, phosphorene, stanene, borophene and antimonene [20–25]. Silicon-doped phosphorene significantly enhanced CO adsorption, whereas sulfur-doped phosphorene showed poor interaction with CO with no appreciable charge redistribution. Phosphorene exhibits excellent adsorption capability for H₂S and CH₄, with adsorption distances of 3.25 and 3.52 Å, respectively. The adsorption of these gases causes notable changes in the electrical properties of phosphorene, especially along the armchair direction, and demonstrates high sensitivity and selectivity for trace-level detection of H₂S and CH₄.

For zigzag (ZSbNR) and armchair (ASbNR) antimonene nanoribbons, the NO₂ molecules formed strong chemical bonds upon adsorption, with the NO₂/antimonene adsorption configuration being stable. NO₂ adsorption transforms the electronic structure of antimonene nanoribbons from a narrow-gap to a wide-gap semiconductor. In SnNRs, B–N co-doping significantly enhances adsorption energy and induces a finite band gap at the Γ point.

The gas adsorption characteristics have also been studied for a variety of other nanoribbons synthesized through multi-element doping, such as ASiGeNR, ABNNR, BN, AlN, ZnO, SnSe and GaN. In ASiGeNRs, CO adsorption leads to band gap widening, whereas NO adsorption causes semiconductor-to-metal transition. CO modifies the optical properties, including light absorption and reflection behaviour, whereas NO exhibits a stronger interaction with the SiGe surface, resulting in significant structural distortion and high sensing efficiency [26]. CO adsorption on BN and AlN nanoribbons reduces the band gap and induces magnetic moments [27]. NH₃ adsorption on α-MoO₃ nanoribbons has shown great promise, with sensors capable of detecting NH₃ concentrations as low as 50 ppb and a detection limit of 280 ppt [28]. CO adsorption on zigzag ZnO nanoribbons (zZnONRs) is an exothermic process that alters electronic properties. Interaction with CO molecules changes the electronic and transport characteristics of zZnONRs, whereas NO adsorption significantly affects the electronic behaviour of armchair ZnO nanoribbons, including transitions from direct to indirect band gaps and a shift towards semimetallic or metallic properties [29,30]. For SnSe and GaN nanoribbons, the adsorption of gases such as CO₂, CO, NO₂, NO, O₂ and SO₂ revealed that SnSe strongly adsorbs NO and SO gases, with NO adsorption inducing a magnetic moment. Armchair GaN nanoribbons, which are intrinsically semiconducting, transform into metallic systems upon the adsorption of these gas molecules [31,32].

Recently, significant progress has been made in the theoretical investigation of CO and NO adsorption by nanoribbon systems. For instance, NO adsorption on alkali- and transition-metal-doped GNR has been reported to induce substantial charge transfer and electronic structure modifications, confirming the high sensitivity of such systems to NO molecules [33]. Similarly, density functional theory (DFT) studies of CO adsorption on transition-metal-doped AGNR revealed notable changes in the adsorption energies and electronic characteristics [16]. These recent studies highlight the growing interest in gas–nanoribbon interactions and provide a relevant context for evaluating the novelty of armchair silicon-tin nanoribbons (ASiSnNRs), which have not yet been systematically explored for CO and NO adsorption.

From the above analysis, it is evident that nanoribbon materials hold substantial promise for a wide range of applications. In this study, we focused on ASiSnNRs and their interactions with CO and NO gas molecules. The structural, electronic, optical and adsorption characteristics were comprehensively evaluated to assess their application potential in gas sensing technologies.

## 2. Model and computational method

The unit cell of the ASiSnNR consists of six silicon (Si) atoms, six tin (Sn) atoms and four hydrogen (H) atoms passivating the ribbon edges to enhance structural stability. The unit cell dimensions along the *x*-, *y*- and *z*-axes were set to 20.000, 25.500 and 8.486 Å, respectively, to ensure periodic boundary conditions for the supercell configuration. To avoid spurious interactions between periodic images, vacuum layers of 20 Å along the *x*-direction and 25.5 Å along the *y*-direction were employed. Additional test calculations with larger vacuum dimensions confirmed that the adsorption energies varied negligibly, validating that the chosen supercell size was sufficient for reliable simulation of adsorption processes. Based on this structure, two gas adsorption configurations were constructed and optimized: one with CO (denoted as ASiSnNR–CO) and the other with NO (denoted as ASiSnNR–NO). All calculations were performed using the Vienna *ab initio* simulation package [16,33], based on DFT. The electron exchange–correlation interactions were treated using the generalized gradient approximation (GGA) with the Perdew–Burke–Ernzerhof (PBE) functional [34] and projector-augmented wave method [35]. To validate the electronic band structure obtained from PBE, additional calculations were performed using the Heyd–Scuseria–Ernzerhof (HSE06) hybrid functional [36]. The wave functions were expanded using a plane-wave basis set with a kinetic energy cut-off of 350 eV. A 1 × 1 × 8 Monkhorst–Pack k-point grid was used for Brillouin zone sampling in the structural optimization. The convergence criteria were set to EDIFF = 10⁻⁸ eV for the total energy between successive ionic steps, and EDIFFG = −10⁻⁸ eV Å^−1^ for the Hellmann–Feynman force threshold. After structural optimization, self-consistent field calculations were performed, and the structural, electronic and optical properties were computed and analysed.

## 3. Results and discussion

### 3.1. Structural characteristics

To evaluate the stability of the investigated structures, the cohesive energy of the pristine ASiSnNR was calculated along with the adsorption energies of the ASiSnNR–CO and ASiSnNR–NO configurations. Cohesive energy was computed using the following equation [37]:


(3.1) $$E_{coh}=\;\frac{E_{SiSnNR}-\left(E_{Si}+E_{Sn}\right)}{n},$$

where $E_{coh}$ is the cohesive energy, $E_{SiSnNR}$ is the total energy of the ASiSnNR structure, $E_{Si}$ and $E_{Sn}$ are the free energies of the isolated Si and Sn atoms, respectively, and *n* is the total number of atoms in the system. The calculated cohesive energy is approximately −1.03 eV. This value indicates strong bonding interactions between the Si and Sn atoms within the crystal lattice, confirming that the ASiSnNR is thermodynamically stable and can exist under ambient or experimental conditions. The adsorption energy was calculated using the following equation [38]:


(3.2) $$E_{A}=\;E_{t}-E_{n}-\;E_{g},$$

where $E_{A}$ is the adsorption energy, $E_{t}$ is the total energy of the nanosheet after gas adsorption, $E_{n}$ is the energy of the pristine nanosheet and $E_{g}$ is the energy of the isolated gas molecule. The calculated adsorption energies are approximately −0.01 eV for CO and −0.68 eV for NO. These results indicated that CO interacted very weakly with the ASiSnNR surface, suggesting a physisorption mechanism dominated by van der Waals forces. In this case, the CO molecule can easily desorb back into the environment, rendering it unsuitable for CO gas-sensing applications owing to its poor gas-retention capability. By contrast, the larger magnitude of the adsorption energy for NO implies a chemisorption process, where charge transfer between the NO molecule and the ASiSnNR surface is likely to occur, potentially accompanied by a slight local structural reconstruction at the adsorption site. This interaction was sufficiently strong to stably anchor NO molecules to the surface without causing molecular dissociation or disrupting the lattice structure of the material. These findings suggest that ASiSnNRs may serve as promising candidates for NO gas sensing, where adsorption-induced electronic or structural changes can be exploited for detection.

**Figure 1.**
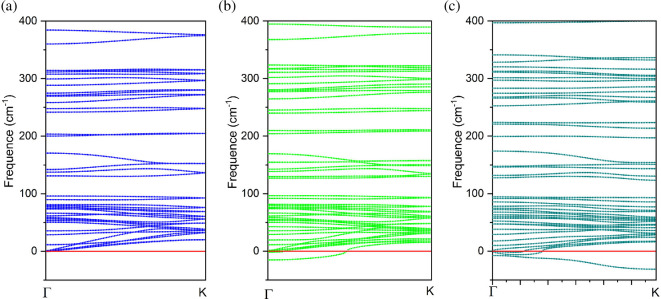
Phonon band structure of (a) ASiSnNR, (b) ASiSnNR–CO, and (c) ASiSnNR–NO.

To evaluate the dynamic stability of the structures, the phonon band structures of ASiSnNR, ASiSnNR–CO and ASiSnNR–NO were calculated. Figure 1a–c illustrates the phonon dispersion curves of pristine ASiSnNR and ASiSnNR after CO and NO adsorption, respectively.

The phonon spectrum of pristine ASiSnNR exhibited no imaginary (negative frequency) modes, indicating that the structure was dynamically stable. Upon CO adsorption, slight modifications were observed in the phonon dispersion; however, no significant imaginary modes were observed. This suggests that the structure remained dynamically stable, consistent with the low adsorption energy and relatively weak interaction between CO and the ASiSnNR surface, which did not lead to notable structural deformation. By contrast, NO adsorption results in the emergence of more imaginary modes in the phonon spectrum, implying a potential reduction in the dynamic stability. This observation aligns with the higher adsorption energy of NO, which indicates that the molecule may induce localized structural reconstruction or lattice softening. Nonetheless, the instability is modest and the system remains sufficiently robust for sensing applications. The imaginary phonon modes appearing after NO adsorption are localized and of small magnitude and thus do not significantly compromise the overall dynamical stability of the ASiSnNR system. Similar behaviour has been reported in previous studies, for instance, in NO adsorption on MoSi₂N₄ [39] and on Sn-based nanoribbons [40], where strong chemisorption induces local lattice softening leading to the emergence of such imaginary modes. In our case, these features are consistent with the strong interaction between NO and the nanoribbon surface, which is also reflected in the electronic structural transition from semiconductor to metal.

**Figure 2.**
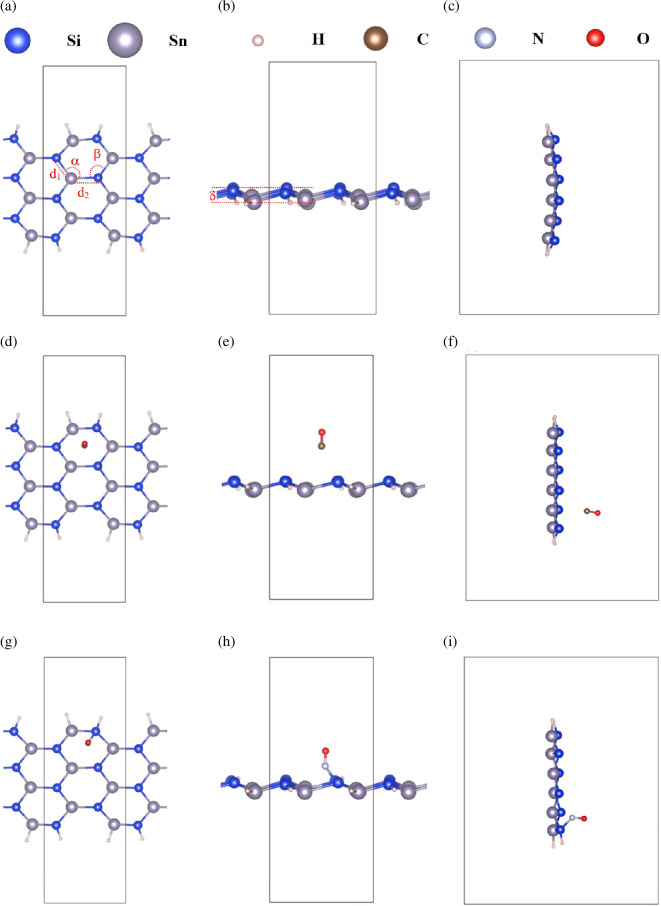
Structures of (a–c) ASiSnNR, (d–f) ASiSnNR–CO, and (g–i) ASiSnNR–NO.

The fundamental structural parameters of the systems were analysed to evaluate the structural changes before and after gas adsorption. Figure 2 illustrates the atomic structures of ASiSnNR, ASiSnNR–CO and ASiSnNR–NO after adsorption. Figure 2a–c shows the pristine ASiSnNR structure, figure 2d–f shows the configurations of ASiSnNR–CO, and figure 2g–i shows those of ASiSnNR–NO.

Table 1 summarizes the key structural parameters, including bond lengths (d₁, d₂), bond angles and buckling values. These parameters provide insight into how gas adsorption affects the local geometry of nanoribbons, revealing whether adsorption induces significant structural deformation or preserves the original lattice integrity.

**Table 1.** The fundamental structural parameters.

configuration	d_1_ [Si–Sn] (Å)	d_2_ [Si–Sn] (Å)	*α* (°)	*β* (°)	*δ* (Å)
ASiSnNR	2.62534	2.84358	119.51	118.89	1.09883
ASiSnNR–CO	2.62221	2.88128	118.76	119.54	0.73868
ASiSnNR–NO	2.63259	2.75085	121.33	124.49	0.90608

The results showed that the pristine ASiSnNR structure exhibited the highest buckling, approximately 1.1 Å, indicating a characteristic distortion arising from the alloying of Si and Sn atoms. The Si–Sn bond lengths in the pristine configuration were non-uniform, probably owing to edge effects or local asymmetry induced by the armchair geometry. After CO adsorption, buckling was significantly reduced (*δ* = 0.73868 Å), suggesting slight planarization of the structure. This implies that the interaction between the CO molecule and the surface was relatively weak, causing minor structural modifications without substantial deformation.

The slight variations observed in the bond lengths and angles further support the conclusion that CO adsorption proceeds via weak physisorption, which aligns well with the previously calculated low adsorption energy. By contrast, NO adsorption results in a buckling value that remains lower than that of the pristine case but higher than that of the CO-adsorbed system, indicating a stronger interaction than CO, yet not strong enough to disrupt the structural framework. The *α* and *β* bond angles increased significantly, reflecting notable lattice distortion, which is a hallmark of chemisorption.

These observations suggest that CO interacts weakly with the surface, making it suitable for CO-sensing applications owing to its reversibility and ease of desorption. However, the stronger interaction with NO, characterized by more pronounced structural distortions, indicates potential applications in catalysis and in the capture and mitigation of toxic NO gas.

### 3.2. The charge density distribution

**Figure 3.**
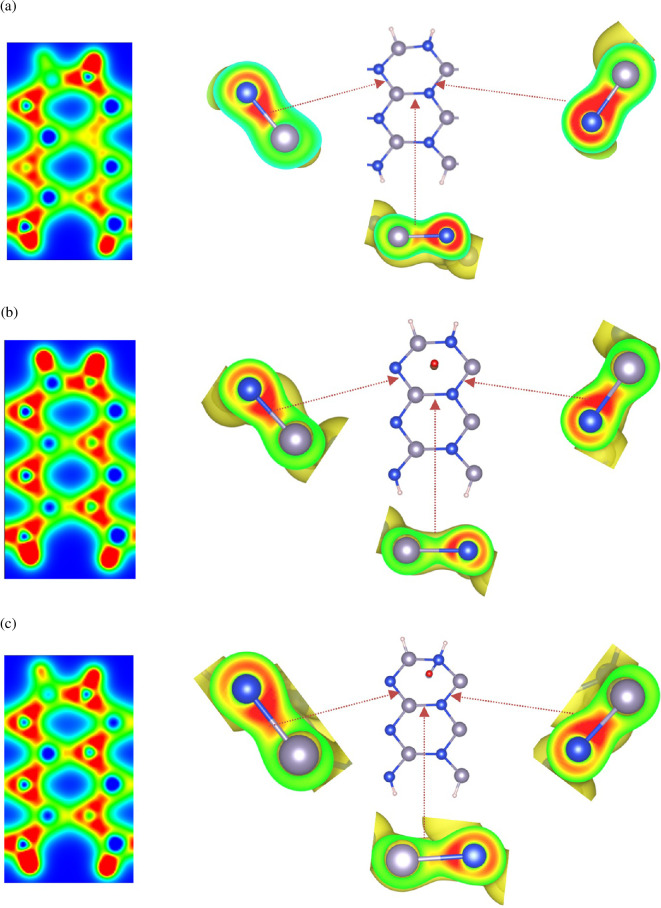
Charge density distribution of (a) ASiSnNR, (b) ASiSnNR–CO, and (c) ASiSnNR–NO.

Figure 3 shows the charge density distribution across the entire surface and around the hexagonal rings of the pristine ASiSnNR (figure 3a), ASiSnNR–CO (figure 3b) and ASiSnNR–NO (figure 3c). In the pristine structure, regions of high charge concentration (represented in red and yellow) are primarily located between the Si and Sn bonds and at the centres of the hexagonal rings. The charge density was evenly distributed around the atoms and bonds, indicating a stable and symmetric structure without significant polarization. This suggests strong covalent bonding between the Si and Sn atoms with relatively uniform and symmetric charge sharing.

Upon gas adsorption, the charge distribution becomes more localized, particularly around the adsorption sites. No strong chemical bonds were formed during the CO adsorption. The local charge density changes only slightly, predominantly around the oxygen atom, which tends to withdraw electron density from the contact region. This indicates that CO adsorption induced minimal and localized charge redistribution, confirming the physical nature of the interaction. The overall lattice remained largely unperturbed, and the charge distribution retained its symmetry.

By contrast, NO adsorption led to more pronounced changes in the charge density. The bright red and deep yellow regions emerge, especially at the NO–ASiSnNR interface. Significant charge accumulation occurs in the vicinity of the interaction zone, with clear evidence of charge transfer between the nitrogen atom and nearby Si or Sn atoms. These strongly polarized regions indicate the formation of covalent bonds and substantial charge redistribution, which are hallmarks of chemisorption.

This analysis of the charge density before and after adsorption revealed that CO weakly interacted with the surface, inducing only minor perturbations in the charge distribution. This behaviour is desirable for gas-sensing applications where rapid desorption and reversibility are required. By contrast, NO interacts more strongly, inducing strong polarization and real charge transfer and forming chemical bonds with the surface. These features make ASiSnNR a suitable candidate for NO capture and the development of highly sensitive NO sensors.

### 3.3. Elecronic band structure—density of states

To evaluate the electronic properties of the investigated systems, their electronic band structures and projected density of states (PDOS) were analysed comprehensively. Table 2 summarizes the calculated band gap values (*E*_g_) of the pristine and gas-adsorbed ASiSnNR structures using two different methods: the GGA with the PBE (GGA-PBE) functional and the more accurate hybrid Heyd–Scuseria–Ernzerhof (HSE06) functional.

**Table 2.** Band gap of structures.

configuration	*E*_g_ (eV) (GGA-PBE)	*E*_g_ (eV) (HSE06)
ASiSnNR	0.4341	0.5887
ASiSnNR–CO	0.4676	0.6322
ASiSnNR–NO	0.0036	0

**Figure 4.**
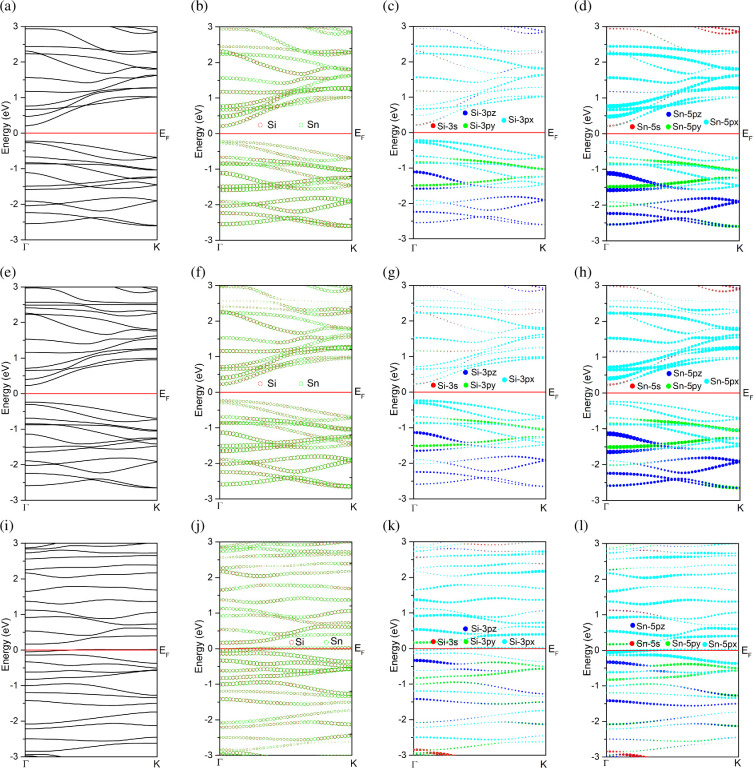
The electronic band structures of the pristine and gas-adsorbed systems: (a–d) ASiSnNR, (e–h) ASiSnNR–CO, and (i–l) ASiSnNR–NO.

Figure 4 shows the electronic band structures of pristine and gas-adsorbed systems. Figure 4a shows the overall band structure of ASiSnNR, and figure 4b shows the atomic contributions from the Si and Sn atoms. Figure 4c,d illustrates the orbital-resolved contributions of the Si and Sn atoms decomposed into s, pₓ, p_y_ and p_z_ orbitals, respectively. Figure 4e–l shows the corresponding data for the ASiSnNR–CO and ASiSnNR–NO configurations, respectively.

**Figure 5.**
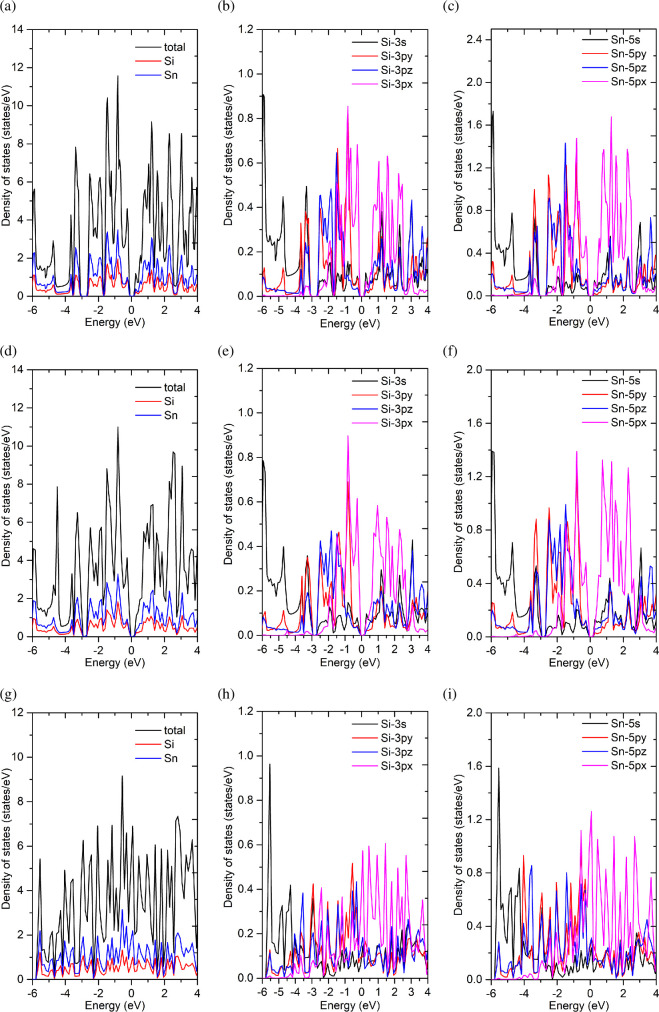
The density of states (PDOS) for the pristine and gas-adsorbed structures: (a–c) ASiSnNR, (d–f) ASiSnNR–CO, and (g–i) ASiSnNR–NO.

Figure 5 illustrates the PDOS of pristine and gas-adsorbed structures. Figure 5a shows the total and PDOS of Si and Sn for pristine ASiSnNR, whereas figure 5b,c shows the orbital-resolved PDOS for each element. Similarly, figure 5d–i presents the orbital contributions of the ASiSnNR–CO and ASiSnNR–NO systems, respectively.

The results show that for the pristine ASiSnNR structure, the band gap calculated using the GGA-PBE method was approximately 0.4341 eV, indicating narrow-gap semiconducting behaviour. The HSE06 hybrid functional yielded a slightly larger band gap of 0.5887 eV, which is generally considered to be in better agreement with the experimental values than GGA-PBE. This suggests that ASiSnNRs have potential applications in nanoscale electronic devices, particularly field-effect transistors and gas sensors.

Upon NO adsorption, the band gap approached zero under both calculation schemes, indicating a transition to metallic behaviour. This implies that NO adsorption leads to significant electronic reconstruction, eliminating the band gap and resulting in strong chemisorption. The system becomes highly conductive, which is particularly favourable for gas-sensing applications, as even small amounts of NO can induce a drastic change in the electrical conductivity.

The band structure analysis confirmed these findings. Prior to gas adsorption (figure 4a–d), the material displayed semiconducting characteristics with a narrow band gap (0.43 eV), and no conduction bands intersected the Fermi level. Atomic and orbital contributions (figure 4b–d) show that both Si and Sn atoms contribute significantly to the electronic states, particularly through the 3p orbitals of Si and the 5p orbitals of Sn. Among them, the p_z_ orbitals are perpendicular to the plane of the ribbon and dominate near the Fermi level, suggesting their key role in the potential interactions with gas molecules.

After CO adsorption (figure 4e–h), the band structure remained largely unchanged with a slight increase in the band gap. A minor reduction in the Sn contribution near the Fermi level was observed, indicating weak charge redistribution. However, no new states appeared within the band gap, and the p_z_ orbitals continued to dominate both the conduction and the valence bands. These features indicate weak surface interactions, probably physisorption or weak chemisorption. These properties are advantageous for stable and reversible CO sensing, enabling quick recovery and linear response.

By contrast, NO adsorption (figure 4i–l) induces a dramatic transformation in the electronic band structure. The Fermi level crossed the conduction band, resulting in a semiconductor-to-metal transition. The strong contribution of Sn atoms near the Fermi level, along with the significant changes in the p_z_ orbital behaviour, reflects the strong interaction between NO and the nanoribbon surface, which is characteristic of chemisorption. NO is likely to accept electrons from ASiSnNR or form covalent bonds with surface Sn or Si atoms, thereby disrupting the pristine electronic structure.

This abrupt shift in electronic conductivity upon NO adsorption highlights the high sensitivity of ASiSnNR for NO detection, even at trace concentrations. Overall, the band structure and orbital-resolved analysis demonstrated that ASiSnNR could effectively distinguish between CO and NO based on the degree of electronic perturbation upon adsorption. The mild changes caused by CO and the strong alterations induced by NO suggest that this material holds promise for highly selective and sensitive gas-sensing applications.

In a recent study on NO gas adsorption on MoSi₂N₄ monolayers [39], it was observed that the material underwent a transition from an indirect to a direct band gap upon adsorption, accompanied by significant narrowing of the gap. This behaviour closely resembles the present findings shown in figure 4i–l, where the Fermi level crosses the conduction band, resulting in a complete band gap closure (*E*_g_ = 0 eV). Such strong modification of the electronic structure owing to NO adsorption facilitates efficient charge transfer, which is a critical mechanism for gas sensing. Similarly, Hunanyan *et al.* [40] reported that the emergence of new electronic states within the band gap upon gas adsorption is a hallmark of strong interactions between the adsorbate and the surface, leading to enhanced sensor sensitivity. Their study highlighted the importance of the hybridized states formed between the s/p orbitals of the substrate atoms and the orbitals of the gas molecules, which is fully consistent with the orbital-resolved band structure presented in figure 4c–l. Additionally, Jha *et al.* [41] demonstrated the sensing capabilities of indium nitride nanoribbons towards CO, CO₂, NO and NO₂, showing that narrow two-dimensional nanoribbons exhibit high sensitivity and fast response to these gases. The strong band structure alterations observed in this study for both CO and NO suggest that the ASiSnNRs may exhibit similar behaviour, confirming their potential as highly sensitive gas sensing materials.

Density of states (DOS) analysis supported these observations. Pristine ASiSnNR exhibited a narrow band gap, which is consistent with the previously discussed band structure. Both the Si and Sn atoms contribute significantly to the DOS near the Fermi level. For Si, the 3p_y_ and 3p_z_ orbitals dominate near the Fermi level, whereas for Sn, the 5p_y_ and 5p_z_ orbitals are similarly dominant. This indicates that the electronic activity is primarily governed by the hybridized p orbitals of Si and Sn atoms. The DOS spectrum changed significantly. There was an increase in the DOS around the Fermi level, suggesting an enhanced electrical conductivity, which is an encouraging sign for sensing applications. The appearance of new peaks near the Fermi level indicated an interaction between the 3p_z_ orbital of Si and the CO molecule. The increased DOS associated with the 5p_y_ orbital of Sn also suggests hybridization with CO’s electronic state of CO. Following NO adsorption, the DOS at the Fermi level increased significantly, with several peaks intersecting the Fermi energy, confirming the complete closure of the band gap, as observed in the band structure analysis. For Si, the 3p_z_ states were notably intensified and broadened, indicating strong orbital hybridization. For Sn, the 5p states extend into the conduction region, confirming that NO induces a more profound electronic reconstruction than CO. The emergence of new in-gap states further suggests charge exchange between NO and the ASiSnNR surface, which is essential for generating an electrical sensing signal.

In summary, PDOS analysis revealed that hybridization between the p orbitals of Si and Sn with those of CO and NO molecules is the primary mechanism driving the electronic structure modification. The degree of electronic perturbation was substantially greater for NO, which aligns with its higher chemical reactivity. This reinforces the conclusion that ASiSnNR is more responsive and sensitive to NO, making it a promising material for selective, high-performance gas sensing.

### 3.4. Charge density difference

**Figure 6.**
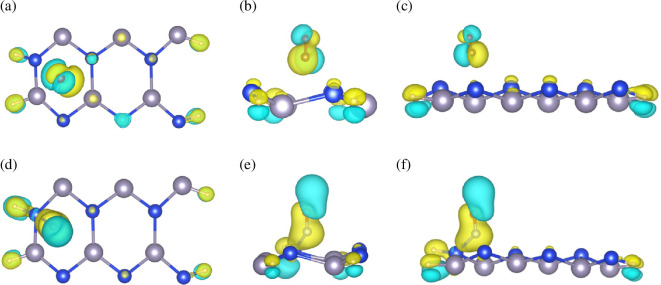
The charge density difference before and after gas adsorption of (a–c) CO, and (d–f) NO.

To gain a more intuitive understanding of the interaction mechanism between the gas molecules and the ASiSnNR surface, particularly the charge transfer process between the adsorbed molecules and the substrate, the charge density difference before and after gas adsorption was analysed. Figure 6 shows the charge density difference (Δρ) maps after CO (figure 6a–c) and NO adsorption (figure 6d–f). In these visualizations, the yellow regions indicate electron accumulation and the blue regions represent electron depletion.

The results show that, following CO adsorption, a distinct region of electron accumulation is observed around the carbon atom of the CO molecule, indicating that CO acts as an electron acceptor and withdraws charge from the ASiSnNR surface. Additionally, an extended charge redistribution was observed between the CO molecule and the adjacent Si or Sn atoms, particularly in the bonding regions, suggesting the formation of weakly hybridized bonds. Although a certain degree of polarization is evident, the extent of charge transfer remains modest, consistent with the DOS results, which reflect only moderate electronic perturbations owing to CO interactions. By contrast, the charge density difference upon NO adsorption revealed significantly stronger charge redistribution. Alternating regions of charge accumulation and depletion were observed around the O and N atoms of the NO molecule as well as across the ASiSnNR surface. This indicated a more directional and intense electronic interaction. Notably, the electron accumulation region spans between the NO molecule and the nearby Si/Sn atoms, suggesting the possible formation of covalent bonds or d–π interactions between the p orbitals of NO and p/d orbitals of the substrate. Furthermore, the charge redistribution extends beyond the adsorption site and into the interior of the nanoribbon lattice, confirming that NO adsorption induces both localized and delocalized electronic structural changes. These observations are in excellent agreement with the previously discussed DOS results, where NO exerts a much stronger perturbative effect on the electronic structure than CO.

In summary, charge density difference analysis revealed that NO possesses a stronger ability to accept and redistribute charge compared to CO, leading to more pronounced electronic effects and greater alterations in the electronic structure. Although both gas molecules exhibit the potential for chemical interaction with the ASiSnNR surface, the degree of interaction differs markedly, enabling the system to exhibit high gas selectivity. The adsorption process is directional and exhibits chemical characteristics rather than a simple case of physical adsorption. This is critical for developing accurate and highly sensitive gas sensors. These findings complement and reinforce the electronic structure and DOS results, collectively confirming that ASiSnNR is a promising candidate material for gas-sensing applications, particularly for the detection of CO and NO.

Compared with previously reported nanoribbon systems, the ASiSnNRs demonstrated unique adsorption-induced modifications in both electronic and optical properties. For instance, in ASiGeNRs, CO adsorption was found to induce a moderate band gap widening, whereas NO adsorption converted the system from a semiconductor to a metallic state [26]. Similarly, ZnONRs exhibit a transition from direct to indirect band gap or even to semimetallic behaviour upon gas adsorption [29,30], and GaNNRs transform from semiconducting to metallic states when exposed to molecules such as CO or NO [31]. By contrast, the ASiSnNRs display two distinctive features: (i) a highly selective semiconductor-to-metal transition triggered specifically by NO adsorption, accompanied by pronounced charge redistribution and lattice distortion; and (ii) a remarkable anisotropic enhancement of optical absorption upon CO adsorption, particularly in the ultraviolet (UV) region. These combined effects have not been simultaneously reported in other nanoribbon systems, highlighting the unique potential of ASiSnNRs for selective and sensitive gas detection.

The theoretical results obtained in this study are also consistent with available experimental findings on related two-dimensional nanomaterials. For instance, experimental studies on graphene-based sensors demonstrated that NO molecules strongly chemisorb on the surface, leading to significant conductance changes, whereas CO interacts only weakly via physisorption, with minimal impact on electronic transport [42–44]. These observations are in excellent agreement with the present results for ASiSnNR, where NO induces a semiconductor-to-metal transition accompanied by strong charge redistribution, whereas CO adsorption only produces minor structural and electronic modifications. Additional insights from defect-engineered graphene further highlight that NO adsorption is intrinsically stronger than CO, which aligns with our phonon and charge density difference analyses [45]. Such consistency with experimental findings reinforces the reliability of the present calculations and underscores the potential of ASiSnNR as a selective and highly sensitive material for gas-detection applications.

### 3.5. Real-space wave function distribution

**Figure 7.**
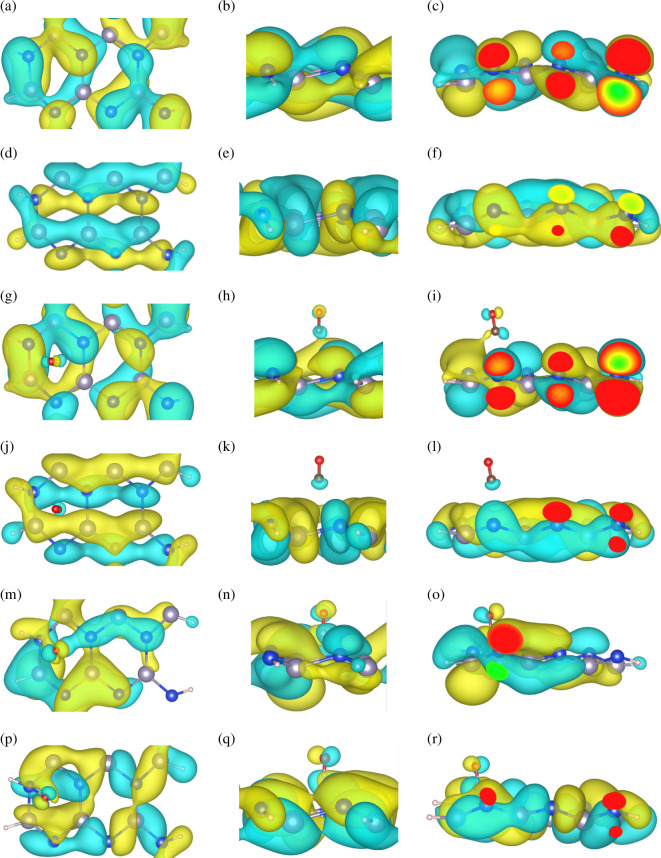
Real-space wave function distribution of (a–f) ASiSnNR, (g–l) ASiSnNR–CO, and (m–r) ASiSnNR–NO.

Figure 7 presents the real-space distribution of the wave functions extracted from the plane-wave coefficients of the Kohn–Sham orbitals at the Γ-point using the data from the WAVECAR file. Figure 7a–c illustrates the conduction band minimum (CBM), and figure 7d–f shows the valence band maximum (VBM) of the pristine ASiSnNR. Figure 7g–l shows the CBM and VBM of the ASiSnNR–CO configuration, respectively, whereas figure 7m–r shows the CBM and VBM of the ASiSnNR–NO structure, respectively.

CBM represents the lowest unoccupied molecular orbital, whereas VBM corresponds to the highest occupied molecular orbital. For the pristine ASiSnNR, both the CBM and VBM wave functions were predominantly localized along the edges of the nanoribbon, with a significant distribution around the Si and Sn atoms. Notably, the CBM exhibited a more delocalized spatial profile, whereas the VBM appeared more localized. This asymmetry suggests that the mobility of charge carriers is higher for electrons than for holes. Upon CO adsorption, the CBM wavefunction exhibited an extended distribution towards the CO molecule, indicating hybridization between the conduction states of the nanoribbon and the p orbitals of the carbon atom in CO. Simultaneously, the VBM became more localized around the oxygen atom, suggesting that CO not only influences the conduction band but also alters the valence band states of the host material. For NO adsorption, the interaction effects are even more pronounced. Both CBM and VBM wave functions displayed broader spatial distributions and were strongly concentrated around the adsorption region. This indicates intense orbital hybridization between the NO molecule and the ASiSnNR substrate, confirming strong electronic coupling and reinforcing the chemisorption character inferred from previous charge density and DOS analyses.

The significant changes observed in the spatial distribution of the wave functions upon gas adsorption, particularly in the case of NO, demonstrate the strong modulation of the electronic band structure. These observations are consistent with the Fermi level shift and transformation of the electronic DOS revealed in the DOS analysis. This result further confirms that ASiSnNR possesses high potential for gas-sensing applications, with its electronic structure being effectively tunable through selective molecular adsorption.

### 3.6. Optical properties

Fundamental optical quantities, including the complex dielectric function, absorption spectrum, reflectivity and transmittance, were investigated to evaluate the optical absorption and reflection characteristics of the material systems. The results indicate that both ASiSnNR and ASiSnNR–CO exhibit partial absorption and reflection of the incident light, whereas ASiSnNR–NO demonstrates nearly 100% transmittance across the investigated spectral range, showing no significant absorption or reflection. This suggests that NO adsorption significantly alters the optical response, potentially leading to transparency of the system.

**Figure 8.**
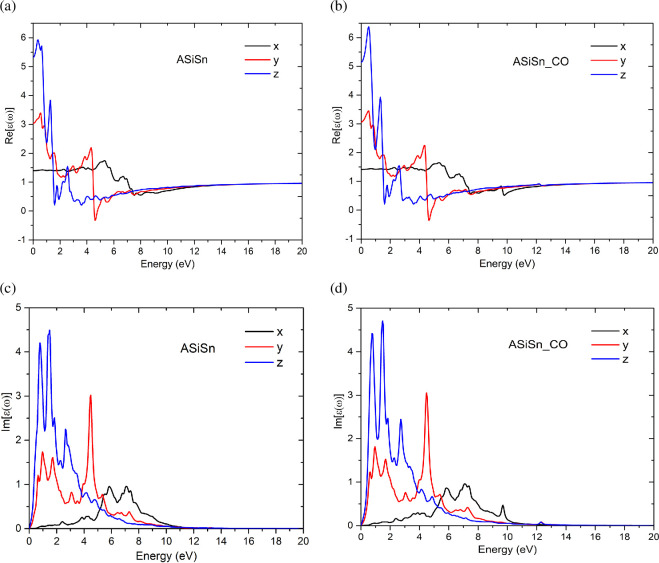
The real (a,b), and imaginary (c,d) parts of the dielectric function.

**Figure 9.**
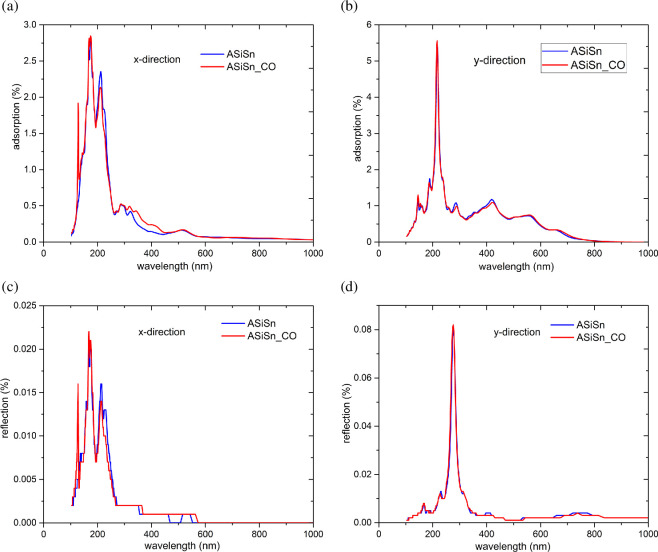
The wavelength-dependent optical absorption and reflection spectra of (a,b) ASiSnNR, and (c,d) ASiSnNR–CO.

**Figure 10.**
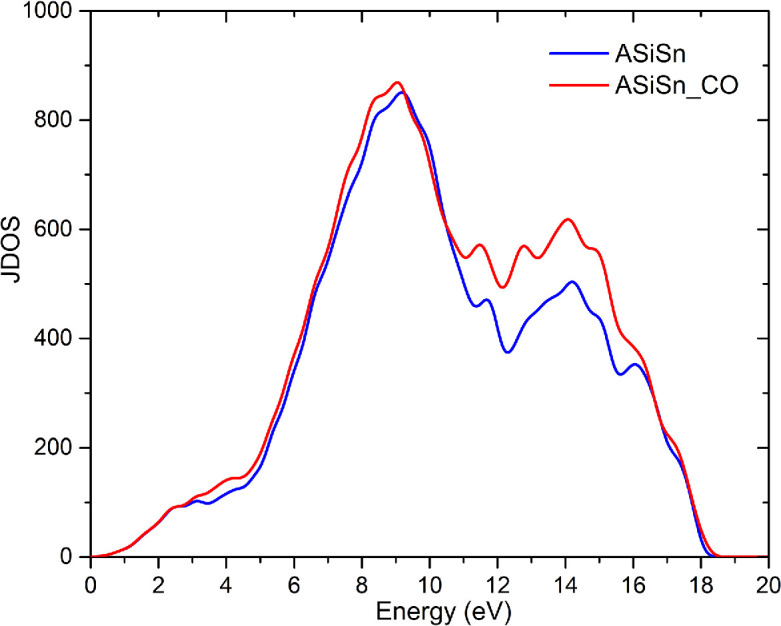
The joint density of states (JDOS) for ASiSnNR and ASiSnNR–CO.

Figure 8 shows the real (figure 8a,b) and imaginary (figure 8c,d) parts of the dielectric function of ASiSnNR and ASiSnNR–CO as a function of the photon energy along the *x*-, *y*- and *z*-polarization directions. Figure 9 illustrates the wavelength-dependent optical absorption (figure 9a,b) and reflection spectra (figure 9c,d) of the ASiSnNR and ASiSnNR–CO. Figure 10 shows the joint density of states (JDOS) for both the structures, providing insight into the available optical transition states.

The results from both the real and imaginary parts of the dielectric function revealed distinct optical anisotropy in both systems, with significant differences observed along the three crystallographic directions. For the pristine ASiSnNR, the real part Re[ε(ω)] exhibited a pronounced peak in the low-energy region (<2 eV) along the *z*-direction, indicating a strong polarizability perpendicular to the nanoribbon plane. Meanwhile, the imaginary part Im[ε(ω)] displays notable absorption peaks at approximately 2 and 4 eV, which are particularly pronounced in the *z*- and *y*-directions, suggesting that the material exhibits strong light absorption in the near-UV range and possesses enhanced optical interactions along these directions. Following the CO adsorption, substantial modifications were observed in the dielectric response. Specifically, Re[ε(ω)] along the *z*-direction shows a slight reduction in the low-energy region, reflecting a decrease in dynamic polarizability owing to the electronic reconstruction induced by CO interaction. Conversely, Im[ε(ω)] exhibited both a redshift and enhancement of the absorption peaks in the 2–6 eV range, especially along the *x*- and *y*-directions. This behaviour is attributed to the increased optical absorption arising from the orbital hybridization between the CO molecule and the ASiSnNR substrate.

The notable changes in the dielectric response upon gas adsorption demonstrate the high optical sensitivity of the ASiSnNR to CO. These findings not only highlight the material’s potential in optoelectronic and photochemical sensing applications but also underscore the importance of molecular adsorption in tuning its electronic and optical properties.

The analysis of the absorption spectra (figure 9a,b) and reflectance spectra (figure 9c,d) as functions of the incident light wavelength along the *x*- and *y*-directions for the ASiSnNR system before and after CO adsorption provides a quantitative understanding of the light–matter interactions and optical modulation induced by the presence of CO molecules. In the absorption spectra, both systems exhibited strong absorption peaks in the UV region, particularly concentrated within the 100–300 nm wavelength range. Along the *x*-direction (figure 9a), the ASiSnNR–CO structure showed enhanced absorption compared to the pristine ASiSnNR, suggesting increased light absorption owing to orbital hybridization between the substrate and the CO molecule. However, in the *y*-direction (figure 9b), the absorption intensity increased only slightly and the spectral shape remained nearly unchanged. This indicates a directionally selective adsorption effect in which CO primarily influences the optical response along the bonding direction.

The reflectance spectra (figure 9c,d) demonstrate that both systems exhibit extremely low reflectivity, particularly in the UV region, with peak reflectance values that do not exceed 0.08% along the *y*-direction. This low reflectance and high absorption make ASiSnNR an excellent candidate for optical applications, such as photodetectors and solar energy harvesting devices. Notably, the changes in reflectance upon CO adsorption were minimal, suggesting that reflection was not the dominant optical mechanism influenced by gas adsorption; rather, absorption played a key role.

Overall, these findings confirm that CO adsorption significantly modulates the absorption spectrum, especially in the UV range and along the direction of direct molecular bonding, highlighting the high optical sensitivity of the ASiSnNR. This further underscores the potential of the material for use in high-performance gas-sensing applications.

Figure 10 illustrates the JDOS of the ASiSnNR nanoribbons before and after CO adsorption. JDOS describes the number of available electronic states that can participate in optical transitions at each energy level, making it a critical factor in determining the optical and electronic behaviour of a material. The JDOS spectra for both systems showed a pronounced increase beginning at approximately 3 eV and reaching a maximum in the 9–13 eV range. Beyond this peak, small oscillations were observed before the JDOS sharply decreased at higher energies (above 17 eV). Notably, the ASiSnNR–CO system (red curve) exhibited a consistently higher JDOS than the pristine ASiSnNR (blue curve) across most energy regions, with a particularly significant increase between 10 and 16 eV. This enhancement indicates that the interaction between the CO molecule and the ASiSnNR framework introduces new electronic states, thereby increasing the probability of electronic transitions between energy levels. The prominent changes in the 10–16 eV range are probably owing to hybridization between the orbitals of the Sn atoms in the ASiSnNR and the π* (antibonding) orbitals of the CO molecule. This resulted in a greater number of accessible electronic states.

Such modifications are of particular importance because they also explain the enhanced optical absorption observed in figure 9a, particularly along the *x*-direction, which is most affected by CO adsorption. Overall, the substantial change in JDOS after CO adsorption highlights the high sensitivity of the system to gaseous species, particularly at the electronic level. These findings further reinforce the potential of ASiSnNRs for use in gas-sensing devices and optoelectronic systems capable of environmental detection via electronic and optical signal variation.

The present results on the adsorption of CO and NO by ASiSnNR can be compared with both theoretical and experimental studies on related low-dimensional nanomaterials. From a simulation perspective, similar work on SiGe nanoribbons reported that CO adsorption only induced a slight widening of the band gap, whereas NO adsorption led to a semiconductor-to-metal transition with significant charge redistribution, consistent with the present findings on SiSnNRs [26]. In addition, BN-doped hydrogenated Sn nanoribbons were predicted to exhibit enhanced CO adsorption owing to strong orbital hybridization [25], while studies on SiNRs demonstrated that the adsorption of CO or NO molecules could substantially modify their electronic structures, particularly through p-orbital hybridization with the host material [46]. These theoretical comparisons collectively support the trend observed here that CO exhibits weak physical adsorption, whereas NO induces chemisorption and strong electronic perturbations.

From an experimental perspective, Sn-containing oxides have been widely studied for gas sensing, and their behaviour provides an indirect validation of the present computational results. For example, Zn_1−x_Sn_x_O thin films demonstrated selective sensitivity towards NO gas, highlighting the role of Sn in stabilizing NO adsorption sites and inducing measurable conductivity changes [47]. Similarly, SnO₂ nanostructures have been experimentally confirmed to exhibit strong responses to NO and NO₂ but only weak responses to CO [48]. These experimental observations are consistent with the present study, where NO adsorption was found to induce significant structural and electronic modifications, whereas CO adsorption remained weak and predominantly physical in nature. Together, the comparison of both the theoretical and the experimental literature strengthens the conclusion that ASiSnNR is a highly promising material for selective and sensitive NO detection.

## 4. Conclusion

This study demonstrates that ASiSnNRs possess significant potential for gas-sensing applications owing to their tunable electronic and optical properties upon molecular adsorption. CO exhibits weak physisorption, induces negligible structural deformation, and results in only a modest enhancement of optical activity. By contrast, NO forms strong chemisorption bonds, triggers a semiconductor-to-metal phase transition and causes a substantial charge redistribution. The involvement of molecular orbitals, particularly the π* orbital of CO and the p orbitals of NO–, in hybridization with surface atoms plays a pivotal role in modulating the electronic band structure and optical response of the system. These findings confirm that ASiSnNR is a highly promising candidate for selective and sensitive gas sensors and is particularly effective for detecting trace concentrations of toxic NO. While the present study establishes the fundamental sensing capabilities of ASiSnNRs towards CO and NO gases, future investigations should extend to broader classes of environmentally relevant and industrial gases, such as NO₂, SO₂, H₂S, NH₃ and volatile organic compounds. A systematic exploration of the adsorption behaviour under varying environmental conditions, such as pressure, temperature and humidity, would provide valuable insights into real-world sensing applications. Furthermore, experimental synthesis and validation of ASiSnNR-based gas sensors are essential for translating theoretical predictions into practical devices. The integration of ASiSnNRs into flexible or wearable platforms could open new avenues for personal health monitoring and industrial safety systems. In particular, the demonstrated selectivity towards NO suggests its potential deployment in medical diagnostics (e.g. monitoring exhaled NO in respiratory diseases) or in detecting pollutant leakage at trace levels. The strong optoelectronic modulation upon adsorption also indicates that the ASiSnNRs may serve as active layers in photoresponsive gas sensors or hybrid devices that combine electronic and optical readouts. Future research should focus on functionalization strategies to further enhance selectivity, reduce the response time and improve stability.

## Data Availability

All data needed to evaluate the conclusions in this paper are available in the Dryad Digital Repository [49].
